# The search for ocean solutions

**DOI:** 10.1371/journal.pbio.3001860

**Published:** 2022-10-17

**Authors:** Nancy Knowlton, Emanuele Di Lorenzo

**Affiliations:** 1 National Museum of Natural History, Smithsonian Institution, Washington, District of Columbia, United States of America; 2 Department of Earth, Environmental and Planetary Sciences, Brown University, Providence, Rhode Island, United States of America

## Abstract

The search for ways to protect and restore ocean health is rapidly accelerating and expanding. A new collection of articles draws on biological and social sciences to suggest changes in how ocean science and conservation are conducted to achieve a sustainable, healthy and inclusive future.

For more than a century, ocean biological research focused on understanding the diversity of marine life and the factors, biological and physical, that shape its patterns of abundance and distribution. As terrestrial creatures, humans were not considered part of the system and were viewed as unable to have an impact on the vast ocean realm, except at very small, near-shore scales. By the last third of the 20^th^ century, however, humans could no longer be ignored. A multitude of studies have now made clear not only the scale of anthropogenic impacts but also the costs of failing to take steps to protect and repair ocean health [1].

Thus increasingly, ocean science has turned to the study of solutions to the problems we have created. Importantly, this endeavor engages both the biological sciences and the social sciences in the broadest sense, including not only how ocean conservation efforts can be optimally designed and implemented, but also how the conduct of research and conservation itself can contribute to a world in which opportunities to engage in ocean science and the benefits of a healthier ocean are equitably shared. This issue of *PLOS Biology* features a collection of contributions on “Ocean solutions for a sustainable, healthy and inclusive future” that touch on this broad array of topics.

Genetic, and now genomic, research has transformed biology across many fields, and marine conservation is no exception. In this collection, van Oppen and Coleman [2] review a multitude of applications ranging from passive monitoring to active intervention, including fisheries stock assessments, detection of invasive and illegally traded species, and the creation of new genotypes better suited to a warmer and more acidic ocean. DNA-based methods are especially powerful when studying microbes and when working in remote locations, as illustrated by Mock et al.’s [3] description of a year-long expedition to the Arctic Ocean in which a team of scientists, joined by journalists and teachers, took samples of sea ice and seawater from a vessel that drifted with the icepack.

A second biological theme explored in this collection concerns marine photosynthetic organisms, which range in size from microalgae to coastal trees. These organisms have a major role in feeding people, supporting biodiversity and slowing climate change because they form the base of the food chain, create the living architecture that shelters an enormous variety of other species, and remove carbon dioxide from the atmosphere. Greene and Scott-Buechler [4] explore the potential for microalgae to transform aquaculture and in the process also reduce carbon emissions. Lovelock et al. [5] review the challenges associated with restoring mangrove forests—vitally important ecosystems that support fisheries, sequester carbon and provide protection against rising seas.

While biological research underpins the advances outlined above, these four articles also point to the importance of societal responses, ranging from permitting and international cooperation to economic incentives and effective land-use planning. They thus provide examples of why the social sciences are increasingly recognized as central to marine conservation success. Three papers in this collection explore this realm by considering the consequences of different assumptions that underpin various conservation approaches. Ota et al. [6] show how five distinct conservation philosophies differ in how they view the interdependence among economic growth, environmental sustainability and social equity, and how their assumptions affect conservation outcomes. Bender et al. [7] argue for a more radical ocean-centric approach, in which the ocean is viewed as a living being with legal rights, and Jacquet and Pauly [8] apply a version of this concept to reimagine the concept of sustainable fisheries as one in which fish are more co-inhabitants than commodities. All three of these commentaries highlight the centrality of indigenous and local communities in ocean conservation, a topic that will be further discussed by Leonard et al. [9] in a forthcoming article.

This brings us to the last two contributions, which address how to broaden the community of people who work on ocean solutions. This is an essential goal that contributes to both environmental justice and better environmental outcomes. The authors of the Consensus View by Satterthwaite et al. [10] are all early career ocean professionals (ECOPs), and they present five actionable pillars that, when enacted, can break down barriers and foster inclusive collaborations. In her Perspective, Moore [11] presents a specific example of how she used a social media campaign, Black in Marine Science, to shine a spotlight on the challenges faced by Black marine scientists, an effort that has grown far beyond its initial scope, and envisions a very different strategy for how to improve the currently very poor diversity and inclusion record in marine science [12].

Like the currents of the ocean itself, ocean solutions and the science that underpins them are on the move. Two recently launched global efforts mentioned in several papers in this collection—the UN Decade of Ocean Science for Sustainable Development and the UN Decade on Ecosystem Restoration —highlight how ocean conservation has captured the world’s attention. Important progress is being made [13], but the challenges remain enormous. We hope this collection gives a sense of the enormous diversity of approaches being explored to meet these challenges, as well as the diversity of individuals doing this essential work, and inspires and informs future efforts.
